# Proportion of Physicians Who Treat Patients With Greater Social and Clinical Risk and Physician Inclusion in Medicare Advantage Networks

**DOI:** 10.1001/jamahealthforum.2023.1991

**Published:** 2023-07-21

**Authors:** Jung Ho Gong, Kenton J. Johnston, David J. Meyers

**Affiliations:** 1 Brown University, Providence, Rhode Island; 2 Washington University in St Louis, St Louis, Missouri

## Abstract

**Importance:**

Medicare Advantage (MA) plans are expanding rapidly, now serving 50% of all Medicare enrollees. Little is known about how inclusion rates of physicians in MA plan networks vary by the social and clinical risks of their patients.

**Objective:**

To examine the association of physicians caring for patients with higher levels of social and clinical risk in traditional Medicare (TM) with the likelihood of inclusion in MA plan networks.

**Design, Setting, and Participants:**

This cross-sectional study evaluated the number of patients of physicians participating in TM Part B in 2019. The data analysis was conducted between June 2022 and March 2023.

**Exposures:**

Quintiles of the proportion of patients who were dually eligible for Medicare and Medicaid and average beneficiary hierarchical condition category (HCC) score (a measure of a patient’s chronic disease burden that is used in risk adjustment and MA plan payment, where higher scores indicate higher risk) in the Part B TM program.

**Main Outcomes and Measures:**

The main outcomes were the proportion of MA plans and enrollees for which physicians were in network.

**Results:**

The analysis sample included 259 932 physicians billing Medicare Part B in 2019. After adjusting for physician, patient, and county characteristics, physicians with the highest quintile of patients with dual eligibility were associated with a lower likelihood of being included in MA plans and being in network with MA enrollees than the lowest quintile physicians (MA inclusion rate, −3.0% [95% CI, −3.2% to −2.8%]; *P* < .001; in-network enrollee proportion, −6.5% [95% CI, −7.0% to −6.0%]; *P* < .001). Similarly, physicians with the highest quintile HCC score were associated with a lower likelihood of being included in MA plans and being in network with MA enrollees than the lowest quintile physicians (MA inclusion rate, −7.5% [95% CI, −7.9% to −7.2%]; *P* < .001; in-network enrollee proportion, −18.7% [95% CI, −19.5% to −18.1%]; *P* < .001). Physicians in medical specialties in the highest clinical risk group (highest quintile HCC score) were associated with a significantly lower likelihood of being in network with MA enrollees than those in the lowest clinical risk group (in-network enrollee proportion, −20.4% [95% CI, −21.1% to −19.8%]; *P* < .001).

**Conclusions and Relevance:**

This cross-sectional study of physicians participating in TM Part B in 2019 found that physicians with higher numbers of patients with social and medical risks in TM were significantly less likely to be associated with MA plans.

## Key Points

**Question:**

Are clinicians who are treating greater numbers of patients with more social and clinical risk factors in traditional Medicare less likely to be included in Medicare Advantage (MA) plan networks?

**Findings:**

In this cross-sectional study of 259 932 clinicians participating in Medicare in 2019, those at the highest quintiles for patients who were dually eligible for Medicare and Medicaid (a proxy measure of social risk) and patients’ hierarchical condition category scores (a proxy measure of clinical risk) were associated with a significantly lower likelihood of being included in MA plan networks and being in network with MA enrollees than those at the lowest such quintiles.

**Meaning:**

Physicians with the highest proportion of patients who were dually eligible for Medicare and Medicaid and with the highest hierarchical condition category scores within traditional Medicare were associated with a significantly lower likelihood of being included in MA networks.

## Teaser

This cross-sectional study examines the association of physicians caring for patients with higher levels of social and clinical risks in traditional Medicare with the likelihood of inclusion in Medicare Advantage plan networks.

## Introduction

The Medicare Advantage (MA) program is a private insurance alternative to the traditional Medicare (TM) program that has been expanding rapidly in the past decade.^1^ The MA program enrollment has more than doubled from 11 million beneficiaries in 2010 to 24 million in 2020 and now enrolls 50% of Medicare beneficiaries.^1^ Medicare Advantage plans are required to provide at least the same services covered by TM but differ in that they can limit their enrollees to a specific network of physicians to control costs and improve quality of care under their capitated payments.^2^ It is not currently known how plans make decisions about what physicians to include in network, and if there are differences in the patients for whom the included physicians care.

There is limited prior evidence on how plans design networks in the MA program. Prior work has found that many MA plans may implement narrow network designs for physician and professional services in Part B. More than a third of MA plans included fewer than 30% of physicians in their contracted counties.^3^ Fewer than 60% of primary care physicians and fewer than 20% of mental and behavioral health clinicians were included in any MA plans in 2019.^4^ At the same time, other work has found that narrow networks may be associated with improvements in plan quality.^5^ Few studies have examined what role physician characteristics play in whether that physician is included in MA plan networks.

Medicare Advantage plan networks are crucial in providing accessible care, but the factors associated with the inclusion of physicians in MA networks are unknown. To maximize risk-adjusted per-enrollee capitated payments, MA plans may limit networks to physicians treating lower numbers of patients with more social and clinical risks who may be viewed as unprofitable. In this study, we sought to assess the association between a physician’s inclusion in MA networks and the number of their patients with social and clinical risks enrolled in TM Part B. Using data on physicians in medical specialties, primary care, and surgical specialties, we aimed to understand (1) whether physicians treating higher numbers of patients with greater social risks in TM are less likely to be included in MA networks, (2) whether physicians treating higher numbers of clinically complex patients in TM are less likely to be included in MA networks, and (3) whether these associations vary by physician specialties and practice locations.

## Methods

### Physician Sample

In this cross-sectional study, the primary study sample was all physicians who treated any TM beneficiaries in Part B in 2019. We used the 2019 Physician Compare data set to identify 3 types of physicians billing Medicare Part B based on their primary specialties^6^: (1) medical specialties (cardiology, endocrinology, gastroenterology, infectious disease, hematology, nephrology, oncology, pulmonology, and rheumatology),^7^ (2) primary care (general practice, family practice, and internal medicine),^4^ and (3) surgical specialties (general surgery, thoracic surgery, colon and rectal surgery, obstetrics and gynecology, neurological surgery, ophthalmology, orthopedic surgery, hand surgery, otolaryngology, plastic and reconstructive surgery, oral and maxillofacial surgery, urology, and vascular surgery).^8^ Nonphysician clinicians were excluded from this study. At the National Provider Identifier (NPI) level, we collected physician sex, physician specialty, physician practice zip code, and year of graduation from medical school. We used the 2019 Medicare Provider Utilization and Payment Data file to collect the number of unique Part B beneficiaries treated, mean beneficiary age, the number of beneficiaries who were dually eligible for Medicare and Medicaid, and mean beneficiary HCC score for each physician. We merged the 2 data sets at the NPI level. Using the definition by the Federal Office of Rural Health Policy,^9^ we determined the rurality of physician practice using the practice zip code. Using the US Department of Housing and Urban county-zip code crosswalk file,^10^ we assigned the physician practice county using the practice zip code. We removed duplicate counties for a given physician practice zip code. We calculated the length of physician clinical practice as the difference between 2019 and the year of graduation from medical school. We calculated the percentage of patients with dual eligibility as the proportion of Part B beneficiaries who were dually eligible for Medicare and Medicaid in 2019. We excluded physicians with missing physician or beneficiary characteristics and limited this analysis to physicians with at least 100 patients to ensure that the estimates were stable.

### Medicare Advantage Data

We used the 2019 Provider Network Data by Ideon, a health technology company that collects MA physician networks linked to individual NPIs and MA contract IDs for 89.2% of all MA beneficiaries in 2019.^11^ We linked these data to publicly available MA plan characteristics files, which included characteristics such as premium and plan rating. We used MA service area files to define in which counties each MA contract is certified to be offered. This study was determined to be exempt by the Brown University institutional review board and follows the Strengthening the Reporting of Observational Studies in Epidemiology (STROBE) reporting guideline. Informed consent was waived because the data were deidentified.

### Primary Outcome

This study’s 2 primary outcomes of interest were the proportion of MA contracts and the proportion of MA enrollees in a physician’s county that were included in network. To calculate the proportion of contracts for each NPI, we first calculated the total number of MA contracts that a given physician was in network for, which serves as the numerator. At the county level, we calculated the total number of MA contracts that operate with at least 1 physician in a given county, which serves as the denominator. Similarly, for the proportion of enrollees in network, we calculated this by dividing the total number of in-network MA beneficiaries at the physician level by the total number of MA beneficiaries in the physician’s practice county. Each physician was assigned to a single practice county on the basis of their NPI practice location. We excluded physicians in counties where there are no MA contracts. See eFigure 1 in Supplement 1 for a visual representation of this process.

### Explanatory Variables

The 2 primary explanatory variables of interest were the proportion of a physician’s patients enrolled in TM Part B that were dually eligible for Medicare and Medicaid and the mean HCC scores of their TM Part B patients. Previous studies have used the proportion of patients dually-eligible for Medicaid as a proxy measure of social risk.^12,13^ Hierarchical condition category (HCC) scores are measures of a patient’s chronic disease burden that the Centers for Medicare & Medicaid Services (CMS) uses in risk adjustment and MA plan payment.^14^ The HCC score was originally designed to adjust capitation payments to MA plans according to beneficiary health risks.^14,15^ We operationalized both variables as quintiles of a physician’s patients with dual eligibility and their mean HCC scores.

### Statistical Analysis

First, we compared the characteristics of physicians across the quintiles of patients with dual eligibility and quintiles of mean beneficiary HCC score. To better understand the rates of inclusion across the distribution of patient dual eligibility and HCC score, we graphed the proportions of in-network MA plans and MA enrollees stratified by each specialty type of physician. Next, using data at the NPI physician level, we fit 2 primary model specifications.

#### Social Risk Model

In the first social risk model, the primary exposure of interest was the physician’s quintile of patients with dual eligibility. We estimated the model using linear regression for each of the 2 outcomes (MA network inclusion rate and MA enrollee proportion) and adjusted for physician practice rurality, gender, specialty, years of service, number of unique beneficiaries, mean beneficiary age, and, importantly, the mean beneficiary level HCC score. We also included physician practice county fixed effects to more directly compare physicians who practiced in the same location with one another and to adjust for market supply of physicians and county MA penetration rate.

#### Clinical Risk Model

In the second clinical risk model, the primary exposure of interest was the quintile of patients’ HCC scores per physician. We also estimated this model using linear regression and included a similar set of covariates (for physician practice rurality, gender, specialty, years of service, number of unique beneficiaries, mean beneficiary age) and practice county fixed effects. Differently from the social risk model, we also adjust for the proportion of beneficiaries who were dually eligible for Medicaid as an adjustment variable.

After estimating the social risk and clinical risk models for both outcomes, we conducted further analysis stratifying each model by specialty type to compare if network decisions appear to be made differently for different types of physicians. Second, we stratified by rurality, as network adequacy standards may be more binding in rural areas. Third, because variables, such as years of practice and rurality, may be mediators of the association of interest, we estimated additional models excluding these controls. Fourth, we tested the inclusion of physicians with different minimum counts of beneficiaries to see how sensitive the results were to the 100-patient cutoff we used. Last, we also tested the outcome of inclusion in no MA networks as an additional sensitivity check. All statistical analyses were performed with Stata statistical software, version 15 (StataCorp LLC) and R statistical software, version 4.0.3 (R Project for Statistical Computing). All analyses used 2-tailed significance tests with α = .05 and robust standard errors. The data analysis was conducted between June 2022 and March 2023.

## Results

The final analysis cohort included 259 932 physicians. A sample inclusion flowchart is in eFigure 2 in Supplement 1. The mean MA inclusion rate was 19.7%. Physicians in the highest quintile of patients with dual eligibility were more likely to be female (17 679 [34.0%] vs 13 447 [25.9%]), primary care physicians (30 835 [59.3%] vs 24 262 [46.7%]), and practice in rural areas (5333 [10.3%] vs 3261 [6.3%]) than those in the lowest quintile (Table 1). Physicians in the highest quintile of mean beneficiary HCC score were less likely to be female (16 460 [31.7%] vs 18 995 [36.5%]), primary care physicians (26 495 [51.0%] vs 31 845 [61.2%]), and practice in rural areas (2287 [4.4%] vs 7500 [14.4%]).

**Table 1.** Characteristics of Physicians by MA Inclusion Rates

Variable	Patients with dual eligibility, %		Mean beneficiary HCC score	
	Quintile 1 (0-10.0)	Quintile 5 (>39.2)	Quintile 1 (0.45-1.14)	Quintile 5 (>2.43)
No.	51 986	51 980	51 993	51 982
Mean MA inclusion rate (SD), %	25.1 (17.6)	17.1 (16.4)	26.0 (19.0)	15.3 (14.4)
Mean in-network enrollee proportion (SD), %	52.9 (29.8)	39.4 (30.6)	52.8 (30.3)	35.7 (30.7)
Sex, No. (%)				
Female	13 447 (25.9)	17 679 (34.0)	18 995 (36.5)	16 460 (31.7)
Male	38 539 (74.1)	34 301 (66.0)	32 998 (63.5)	35 522 (68.3)
Physician specialty, No. (%)				
Medicine	7571 (14.6)	10 384 (20.0)	1501 (2.9)	18 437 (35.5)
Primary care	24 262 (46.7)	30 835 (59.3)	31 845 (61.2)	26 495 (51.0)
Surgery	20 153 (38.8)	10 761 (20.7)	18 647 (35.9)	7050 (13.6)
Physician practice rurality, No. (%)				
Urban	48 725 (93.7)	46 647 (89.7)	44 493 (85.6)	49 695 (95.6)
Rural^a^	3261 (6.3)	5333 (10.3)	7500 (14.4)	2287 (4.4)
Mean practice year (SD), y^b^	27 (11)	23 (13)	26 (12)	20 (12)
Mean beneficiary age (SD), y	74 (2)	69 (6)	72 (4)	72 (5)
Mean No. of unique beneficiaries (SD)^c^	537 (461)	270 (302)	375 (402)	353 (320)
Mean patients with dual eligibility (SD), %	6.1 (2.7)	55.7 (13.9)	13.4 (13.0)	39.6 (16.9)
Mean beneficiary HCC score (SD)	1.20 (0.33)	2.50 (1.27)	1.00 (0.10)	3.31 (1.03)

Abbreviations: HCC, hierarchical condition category; MA, Medicare Advantage.

^a^ Physician practice rurality was determined by the practice zip code using the definition by the Federal Office of Rural Health Policy.

^b^ Years in practice indicate the time between graduation from medical school and 2019.

^c^ Number of unique beneficiaries indicates the number of Medicare Part B beneficiaries treated by a given physician.

In the Figure, the proportions of MA networks and enrollees a physician is in network for are compared with the physicians’ proportion of patients with dual eligibility and with the patients’ mean beneficiary HCC score. As the proportion of patients with dual eligibility and mean HCC scores increase, the likelihood of being included in an MA network and in-network enrollee proportion decreased.

**Figure.**
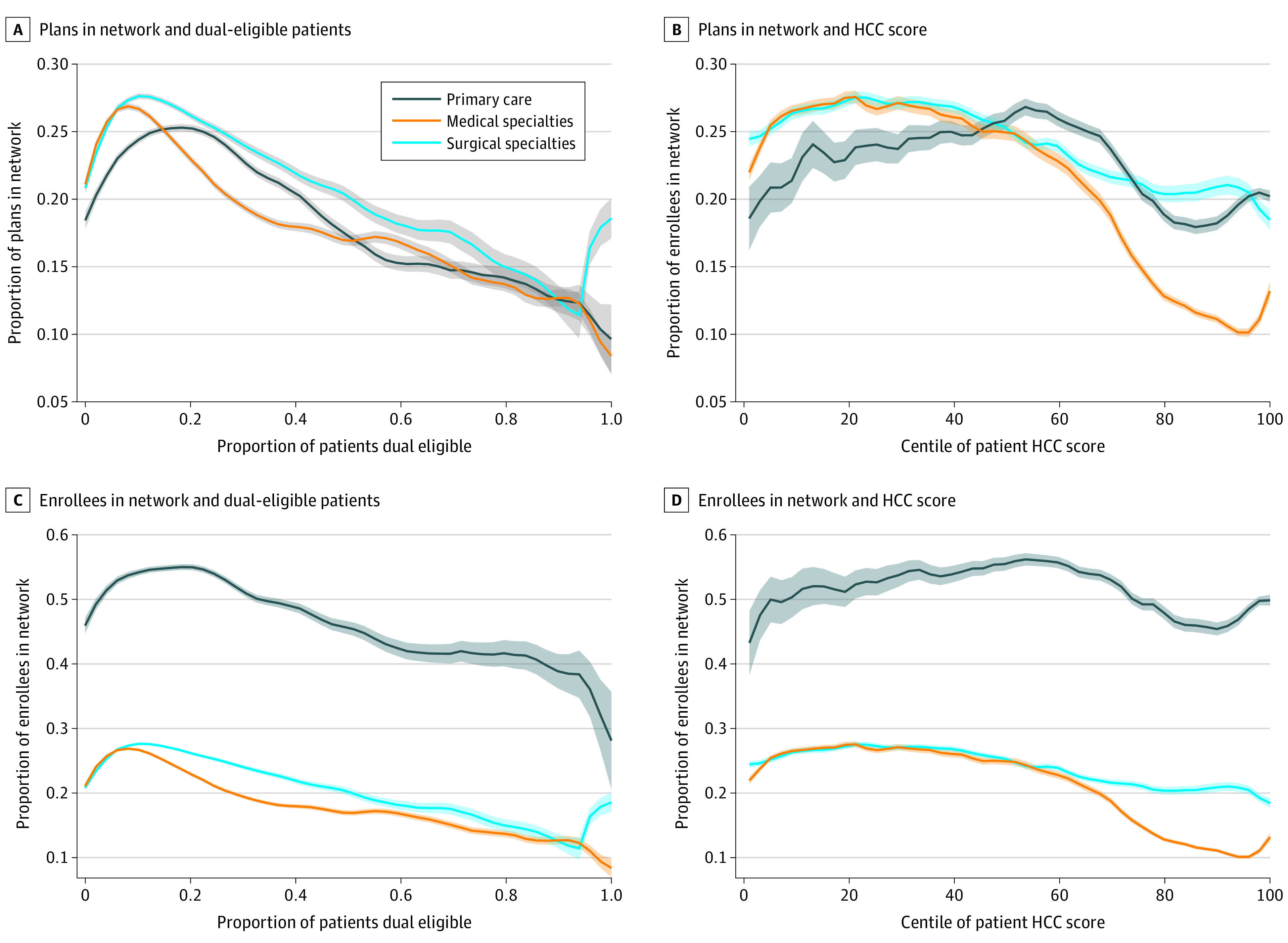
Unadjusted MA Inclusion Rates by Patient Social and Clinical Risks Across Specialties The proportions of MA networks and enrollees a physician are in network for is compared with the physicians’ proportion of patients with dual eligibility (a proxy for social risk) and with the patients’ mean beneficiary HCC score (a proxy for clinical risk; a measure of a patient’s chronic disease burden that is used in risk adjustment and MA plan payment, where higher scores indicate higher risk). As the proportion of patients with dual eligibility and mean HCC scores increase, the likelihood of being included in an MA network and in-network enrollee proportion decreased. Abbreviations: MA, Medicare Advantage; HCC, hierarchical condition categories.

After adjusting for physician and beneficiary characteristics and county fixed effects, we find that physicians in the highest quintile of patients with dual eligibility were associated with lower MA inclusion rates and in-network enrollee proportions compared with physicians in the lowest quintile of patients with dual eligibility (MA inclusion rate, −3.0 percentage point (pp) [95% CI, −3.2 pp to −2.8 pp]; *P* < .001; in-network enrollee proportion, −6.5 pp [95% CI, −7.0 pp to −6.0 pp]; *P* < .001) (Table 2).

**Table 2.** Association of Patient Dual Eligibility and Social Risks With MA Inclusion Rate and In-Network Enrollee Proportion

Patients with dual eligibility^a^				Mean beneficiary HCC score^b^			
Quintile (range), %	Unadjusted (95% CI), %^c^	MA inclusion rate (95% CI), pp	*P* value	Quintile (range), %	Unadjusted (95% CI), %^c^	MA inclusion rate (95% CI), pp	*P* value
**Outcome: MA inclusion rate**							
Q1 (0 to 10.0)	25.1 (25.0 to 25.3)	25.1 [Reference]	NA	Q1 (0.45 to 1.14)	26.0 (25.8 to 26.1)	26.0 [Reference]	NA
Q2 (10.1 to 17.4)	26.1 (26.0 to 26.3)	−0.01 (−0.02 to 0.0)	.25	Q2 (1.15 to 1.37)	26.7 (26.6 to 26.9)	−0.1 (−0.3 to 0)	.08
Q3 (17.5 to 26.2)	24.1 (24.0 to 24.3)	−1.1 (−1.3 to −1.0)	<.001	Q3 (1.38 to 1.75)	25.1 (25.0 to 25.3)	−1.0 (−1.2 to −0.8)	<.001
Q4 (26.3 to 39.2)	20.9 (20.7 to 21.1)	−2.8 (−3.0 to −2.6)	<.001	Q4 (1.76 to 2.43)	20.3 (20.1 to 20.4)	−4.6 (−4.9 to −4.4)	<.001
Q5 (>39.2)	17.1 (17.0 to 17.3)	−3.0 (−3.2 to −2.8)	<.001	Q5 (>2.43)	15.3 (15.2 to 15.5)	−7.5 (−7.9 to −7.2)	<.001
**Outcome: in-network enrollee proportion**							
Q1 (0 to 10.0)	52.9 (52.7 to 53.2)	[Reference]	NA	Q1 (0.45 to 1.14)	52.8 (52.5 to 53.0)	[Reference]	NA
Q2 (10.1 to 17.4)	53.6 (53.3 to 53.8)	−0.6 (−0.9 to −0.3)	<.001	Q2 (1.15 to 1.37)	53.7 (53.5 to 54.0)	−0.9 (−1.2 to −0.6)	<.001
Q3 (17.5 to 26.2)	49.4 (49.1 to 49.7)	−2.9 (−3.2 to −2.5)	<.001	Q3 (1.38 to 1.75)	52.2 (51.9 to 52.4)	−3.3 (−3.6 to −2.9)	<.001
Q4 (26.3 to 39.2)	43.0 (42.7 to 43.2)	−6.7 (−7.1 to −6.3)	<.001	Q4 (1.76 to 2.43)	43.9 (43.7 to 44.2)	−11.2 (−11.7 to −10.7)	<.001
Q5 (>39.2)	39.4 (39.1 to 39.7)	−6.5 (−7.0 to −6.0)	<.001	Q5 (>2.43)	35.7 (35.4 to 36.0)	−18.7 (−19.5 to −18.1)	<.001

Abbreviations: HCC, hierarchical condition category; MA, Medicare Advantage; NA, not applicable; pp, percentage point; Q, quintile.

^a^ The social risk model was adjusted for physician characteristics (eg, physician practice rurality, sex, specialty, and years of clinical practice), beneficiary characteristics (eg, number of unique beneficiaries, mean beneficiary age, and HCC score), and physician practice county fixed effects.

^b^ The clinical risk model was adjusted for physician characteristics (eg, physician practice rurality, sex, specialty, and years of clinical practice), beneficiary characteristics (eg, number of unique beneficiaries, mean beneficiary age, and percentage patients with dual eligibility), and physician practice county fixed effects.

^c^ Unadjusted MA inclusion rates were calculated as margins of probability linear regressions between exposure variables and the outcome variable.

Similarly, after adjusting for physician and beneficiary characteristics and county fixed effects, physicians in the highest quintile of HCC score were associated with lower MA inclusion rates and in-network enrollee proportions compared with those in the lowest quintile (MA inclusion rate, −7.5 pp [95% CI, −7.9 pp to −7.2 pp]; *P* < .001; in-network enrollee proportion, −18.7 pp [95% CI, −19.5 pp to −18.1 pp]; *P* < .001) (Table 2).

Medical specialty physicians were associated with the largest differences in MA inclusion rates and in-network enrollee proportions between the top vs bottom quintile of patients with dual eligibility (MA inclusion rate, −7.2 pp [95% CI, −7.6 pp to −7.1 pp]; *P* < .001; in-network enrollee proportion, −4.4 pp [95% CI, −5.1 pp to −3.8 pp]; *P* < .001) as well as between the highest quintile mean beneficiary HCC score and lowest quintile (MA inclusion rate, −8.2 pp [−8.5 pp to −7.9 pp]; *P* < .001; in-network enrollee proportion, −20.4 pp [95% CI, −21.1 pp to −19.8 pp]; *P* < .001) as compared with primary care and surgical specialty physicians (Table 3). When comparing results for primary care physicians, the differences were substantially smaller with a −0.4 pp (95% CI, −0.8 pp to 0 pp) difference in MA inclusion between the top vs bottom quintile for patients with dual eligibility and no significant difference for the in-network enrollee proportion of patients with dual eligibility (0 pp [95% CI, −1.0 pp to 1.0 pp]; *P* = .92). When comparing by top vs bottom quintile of HCC scores, the differences were smaller for both the MA inclusion rate (−1.3 pp [95% CI, −1.9 pp to −0.6 pp]) and the in-network enrollee proportion (4.0 pp [95% CI, −5.4 pp to −2.5 pp]). Surgical specialty physicians had more similar results to primary care physicians.

**Table 3.** Association of Patient Social and Clinical Risks With MA Inclusion Rate and In-Network Enrollee Proportion Across Specialties

Patients with dual eligibility^a^							Mean beneficiary HCC score, pp^b^						
Quintile (range),%	Medical specialty (95% CI), pp	*P* value	Primary care (95% CI), pp	*P* value	Surgical specialty (95% CI), pp	*P* value	Quintile (range), %	Medical specialty (95% CI), pp	*P* value	Primary care (95% CI), pp	*P* value	Surgical specialty (95% CI), pp	*P* value
**Outcome: MA inclusion rate**													
Q1 (0 to 10.0)	22.8 [Reference]	NA	22.5 [Reference]	NA	24.5 [Reference]	NA	Q1 (0.45 to 1.14)	23.5 [Reference]	NA	24.7 [Reference]	NA	24.6 [Reference]	NA
Q2 (10.1 to 17.4)	−1.3 (−1.5 to −1.0)	<.001	0.7 (0.4 to 1.0)	<.001	1.4 (1.2 to 1.6)	<.001	Q2 (1.15 to 1.37)	0.7 (0.4 to 0.9)	<.001	0.5 (−0.2 to 1.1)	.15	0.4 (0.2 to 0.6)	.001
Q3 (17.5 to 26.2)	−4.0 (−4.3 to −3.8)	<.001	0.8 (0.5 to 1.1)	<.001	0.7 (0.4 to 0.9)	<.001	Q3 (1.38 to 1.75)	−0.5 (−0.7 to −0.2)	.002	0.2 (−0.4 to 0.8)	.59	0.9 (0.6 to 1.1)	<.001
Q4 (26.3 to 39.2)	−6.6 (−6.8 to −6.3)	<.001	0.2 (−0.1 to 0.5)	.24	−0.5 (−0.8 to −0.2)	.001	Q4 (1.76 to 2.43)	−5.5 (−5.8 to −5.2)	<.001	−1.4 (−2.0 to −0.8)	<.001	0.1 (−0.2 to 0.4)	.54
Q5 (>39.2)	−7.2 (−7.6 to −7.1)	<.001	−0.4 (−0.8 to 0)	.04	−1.6 (−2.0 to −1.2)	<.001	Q5 (>2.43)	−8.2 (−8.5 to −7.9)	<.001	−1.3 (−1.9 to −0.6)	<.001	−0.8 (−1.1 to −0.4)	<.001
**Outcome: in-network enrollee proportion**													
Q1 (0 to 10.0)	45.8 [Reference]	NA	50.7 [Reference]	NA	52.4 [Reference]	NA	Q1 (0.45 to 1.14)	50.4 [Reference]	NA	54.0 [Reference]	NA	52.9 [Reference]	NA
Q2 (10.1 to 17.4)	−0.9 (−1.4 to −0.4)	<.001	1.5 (0.8 to 2.2)	<.001	3.0 (2.5 to 3.5)	<.001	Q2 (1.15 to 1.37)	1.0 (0.5 to 1.4)	<.001	1.2 (−0.3 to 2.6)	0.11	0.5 (0.1 to 1.0)	.03
Q3 (17.5 to 26.2)	−3.7 (−4.2 to −3.2)	<.001	1.6 (0.8 to 2.3)	<.001	1.4 (0.8 to 2.0)	<.001	Q3 (1.38 to 1.75)	−1.2 (−1.7 to −0.6)	<.001	0.1 (−1.2 to 1.5)	0.84	0.8 (0.2 to 1.3)	.005
Q4 (26.3 to 39.2)	−6.1 (−6.7 to −5.6)	<.001	0.1 (−0.7 to 0.9)	.82	−1.1 (−1.8 to −0.4)	.003	Q4 (1.76 to 2.43)	−12.9 (−13.5 to −12.3)	<.001	−3.8 (−5.2 to −2.3)	<.001	−1.3 (−2.0 to −0.7)	<.001
Q5 (>39.2)	−4.4 (−5.1 to −3.8)	<.001	0 (−1.0 to 1.0)	.92	−2.6 (−3.4 to −1.7)	<.001	Q5 (>2.43)	−20.4 (−21.1 to −19.8)	<.001	−4.0 (−5.4 to −2.5)	<.001	−3.5 (4.3 to −2.7)	<.001

Abbreviations: HCC, hierarchical condition category; MA, Medicare Advantage; NA, not applicable; pp, percentage point; Q, quintile.

^a^ The social risk model was adjusted for physician characteristics (eg, physician practice rurality, sex, and years of clinical practice), beneficiary characteristics (eg, number of unique beneficiaries, mean beneficiary age, and HCC score), and physician practice county fixed effects.

^b^ The clinical risk model was adjusted for physician characteristics (eg, physician practice rurality, sex, and years of clinical practice), beneficiary characteristics (eg, number of unique beneficiaries, mean beneficiary age, and percentage patients with dual eligibility), and physician practice county fixed effects.

In additional sensitivity analyses, associated differences in MA inclusion rates and in-network enrollee proportions across patient social and clinical risks were greater among physicians practicing in rural vs urban counties (eTable 1 in Supplement 1). The results were similar when adding and removing covariates from the model (eTable 2 in Supplement 1) and by changing the minimum number of patients for a physician to be included in the analysis (eTable 3 in Supplement 1).

## Discussion

This cross-sectional study has 3 key findings. First, physicians who treat a higher proportion of patients dually eligible for Medicare and Medicaid in TM are substantially less likely to be included as in-network physicians in MA and serve MA beneficiaries. Second, physicians, especially those in medical specialties, treating patients with high clinical risk scores in TM are less likely to be included in MA plan networks and be in network with MA enrollees. Notably, this trend differed from primary care physicians where the differences in inclusion were much smaller. Third, these findings were amplified among physicians of medical specialties and those who practice in rural areas. These findings provide the first evidence that MA plans may limit networks to physicians treating TM patients with lower social and clinical risks. More research is needed to compare the MA with TM patient panels of physicians in both programs.

Patient social and clinical risk factors are important determinants of patient health outcomes.^16,17,18^ According to CMS, 60% of beneficiaries with dual eligibility have multiple chronic illnesses.^19^ As MA plans are paid a capitated amount for each enrollee by CMS,^1,2^ patients who have greater care needs may be costlier for MA plans to manage care for. While CMS does use HCC scores to risk adjust the payments that plans receive,^14,15^ they do not adjust for important patient cognitive, functional, and social risk factors, and plans may still lack sufficient incentives to engage with physicians who see greater numbers of socially and clinically complex patients. For example, prior research indicates that the HCC payment model underpredicts costs of treating patients with dual eligibility by 12%.^20^

The MA penetration among older adult patients with dual eligibility has historically lagged due to the lack of support from states and managed care.^21^ However, a recent study suggests that MA networks are expanding to include more patients with dual eligibility in recent years.^22^ From 2009 to 2018, dual enrollees were one of the most rapidly growing populations in MA.^22^ Similarly, prior work has found that the inclusion of patients with dual eligibility in MA plans has increased over the past 10 years, and many MA plans are launching dual-eligible special needs plans (D-SNPs), a special type of plan that aims to manage the care of patients with dual eligibility. However, the present study finds that despite this growth in the number of patients with dual eligibility, MA plans do not seem to be networking with physicians who treat higher proportions of patients with dual eligibility in TM. If these physicians are more experienced in the care of patients with complex medical and social needs, this could limit the promise of plans such as D-SNPs. Additional work is imperative to determine why physicians treating more socially disadvantaged patients in TM may be excluded from MA networks.

We found that these results are primarily driven by a differential selection of medical specialty physicians across the distribution of patients with dual eligibility and that the differences were much smaller or nonsignificant for primary care physicians. This could be explained by plans selecting primary care physicians who care for patients with more socially and clinically complex patients as beneficiaries with dual eligibility are paid more in risk adjustment. A plan may profit from these risk-adjusted payments if they are then able to use utilization control to keep their spending low. This may differ from medical specialty physicians with higher numbers of patients with dual eligibility, which may have more complicated outcomes without the added benefit of bringing more beneficiaries into the plan. Further research into network design should continue to differentiate between primary care and specialty physician dynamics.

The HCC score is a risk-adjustment tool designed by CMS to adjust capitation payments to MA plans according to beneficiary health risks and costs.^14,15^ As the HCC model was originally intended to promote managed care programs to treat chronically ill patients by providing bonus payments,^15^ higher inclusion of physicians treating patients with high HCC scores was expected. However, the present study’s findings suggest that MA plans may prefer physicians treating healthier patients in the TM program. Medicare Advantage plans may find risk-adjusted payments inadequate and limit their networks accordingly. If actual health care costs projected by HCC scores outweigh their capitation payments during an enrollment year, MA plans may narrow their physician networks in the following year. Similarly, Newhouse et al^23^ suggested that MA plans may select physicians by specific HCC components (ie, diabetes, chronic obstructive pulmonary disease). For example, if reimbursements for treating patients with diabetes are not adequately risk adjusted, MA plans would financially benefit from excluding physicians who treat high numbers of patients with diabetes. Further research is needed to assess the distribution of HCC scores and related comorbidities across MA plans at the patient and physician levels.

An alternative potential explanation for the present study’s findings is that physicians who treat patients with greater social and clinical risks may be choosing to forgo inclusion in MA networks. Several factors may contribute to such a decision. First, if MA plans pay less than TM plans pay for care, then it may be more advantageous for these physicians to primarily focus on patients in the TM program. Medicare Advantage plans may also implement prior authorization requirements and implement other practices that could pose an administrative burden for some physicians, decreasing their attractiveness despite the potential access to a greater array of patients. Regardless of whether plans are actively excluding physicians or physicians are actively avoiding plans, it is clear that physicians who treat patients with greater social risks in TM are less likely to be included in MA networks. An additional potential explanation is that patients with low socioeconomic status may have access to lower-quality physicians, and if lower-quality physicians are excluded from MA plans at higher rates, it could also lead to the present study’s findings. In this descriptive analysis, we are not able to differentiate the causal direction of these associations.

### Limitations

First, this study was cross-sectional, and the results cannot assess the causality of patient social and clinical risks in the MA inclusion rate. Second, we used TM Part B data to measure Medicare-participating physician and beneficiary characteristics aggregated at the physician level and compared this with MA county-level plan and contract data. However, the risk profile of physicians’ TM Part B vs MA patients may differ. Third, the methods assigned physicians to only a single practice county and may not fully reflect physicians who practice in multiple counties or physicians who do not keep their NPI registry updated. Fourth, Ideon data did not include the entire 2019 MA physician network data. However, as almost 90% of all MA enrollees were included in the data set, we were able to analyze data on most of the MA enrollees.^4,11^ Fifth, the Ideon data, while the most commonly used file for network analysis in MA research, is still based on the directories reported by MA plans and may not fully represent the true access to in-network physicians. However, given that this analysis is focused on the physician level, the Ideon data still provide a valuable ability to compare physician-level inclusion in networks. Sixth, as the exposure variables are based on TM data, we are unable to compare outcomes for physicians who only treat MA beneficiaries; however, most physicians who treat MA beneficiaries likely also treat TM beneficiaries.

## Conclusions

In this cross-sectional study, we found that physicians who treat more patients with clinical complexity and dual eligibility in Part B in the TM program were less likely to be included in many MA plan networks. This gap regarding network inclusion may result in physicians who are already underresourced being excluded from plans at higher rates. Alternatively, MA plans may be less attractive to these physicians. As MA plan penetration, particularly among enrollees with dual eligibility, increases in coming years, it will be imperative to ensure that patients have access to physicians who can address their care needs.

## Notes

Supplement 1. **eFigure 1.** Diagram of In-Network Calculation **eFigure 2.** Sample Inclusion **eTable 1.** Association of Patient Dual and Social Risks with MA Inclusion Rate and In Network Enrollee Proportion by Rurality **eTable 2.** Sensitivity Analysis removing potential mediators from the analysis. **eTable 3.** Sensitivity analysis by minimum number of TM beneficiaries

Supplement 2.
Data Sharing Statement
