# Supplementary material for: Proportion of Physicians Who Treat Patients With Greater Social and Clinical Risk and Physician Inclusion in Medicare Advantage Networks
Source: JAMA Health Forum. 2023 Jul 21;4(7):e231991. doi: 10.1001/jamahealthforum.2023.1991 (PMC10362476; doi:10.1001/jamahealthforum.2023.1991)
Supplement: Supplement 1. — eFigure 1. Diagram of In-Network Calculation eFigure 2. Sample Inclusion eTable 1. Association of Patient Dual and Social Risks with MA Inclusion Rate and In Network Enrollee Proportion by Rurality eTable 2. Sensitivity Analysis removing potential mediators from the analysis. eTable 3. Sensitivity analysis by minimum number of TM beneficiaries [file jamahealthforum-e231991-s001.pdf]

Supplemental Online Content

Gong JH, Johnston KJ, Meyers DJ. Proportion of physicians who treat patients with greater social and clinical risk and physician inclusion in Medicare Advantage networks. *JAMA Health Forum.* 2023;4(7):e231991. doi:10.1001/jamahealthforum.2023.1991

eFigure 1. Diagram of In-Network Calculation

eFigure 2. Sample Inclusion

eTable 1. Association of Patient Dual and Social Risks with MA Inclusion Rate and In Network Enrollee Proportion by Rurality

eTable 2. Sensitivity Analysis removing potential mediators from the analysis.

eTable 3. Sensitivity analysis by minimum number of TM beneficiaries

This supplemental material has been provided by the authors to give readers additional information about their work.

© 2023 Gong JH et al. *JAMA Health Forum.*

eFigure 1. Diagram of In-Network Calculation

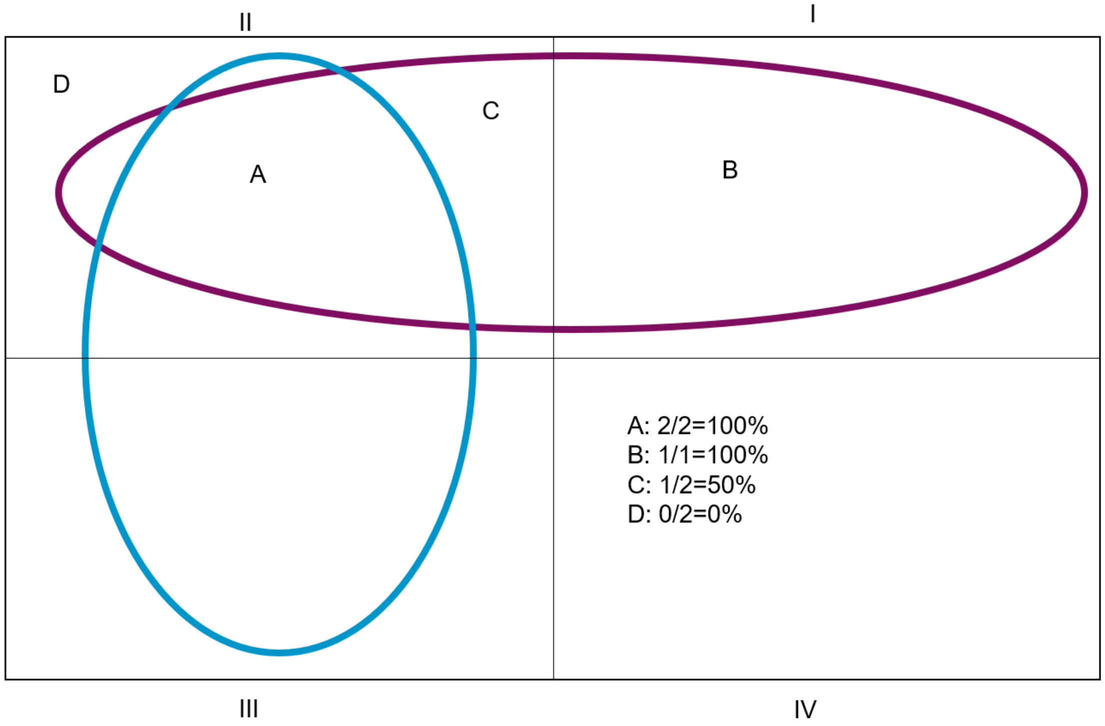

Notes: Each quadrant (I, II, III, IV) represents a county where a physician primarily practices. Each letter (A, B, C, D) represents a physician. The two ovals represent two different MA contracts that have different service areas. To calculate the proportion of contracts each physician was in-network for, the denominator is the number of contracts that operate in that county, and the numerator is the number of contracts the physician is included in. For example, physician A practices in county II. There are two contracts that operate in county II, and physician A is included in both of them so their inclusion proportion is 2/2=100%. In contrast, physician C is

© 2023 Gong JH et al. *JAMA Health Forum.*

only included in one network in county II, so their inclusion proportion is ½=50%. To calculate the proportion on enrollees each physician is included in network for, we weighed the MA contracts by their 2019 enrollment.

© 2023 Gong JH et al. *JAMA Health Forum.*

eFigure 2. Sample Inclusion

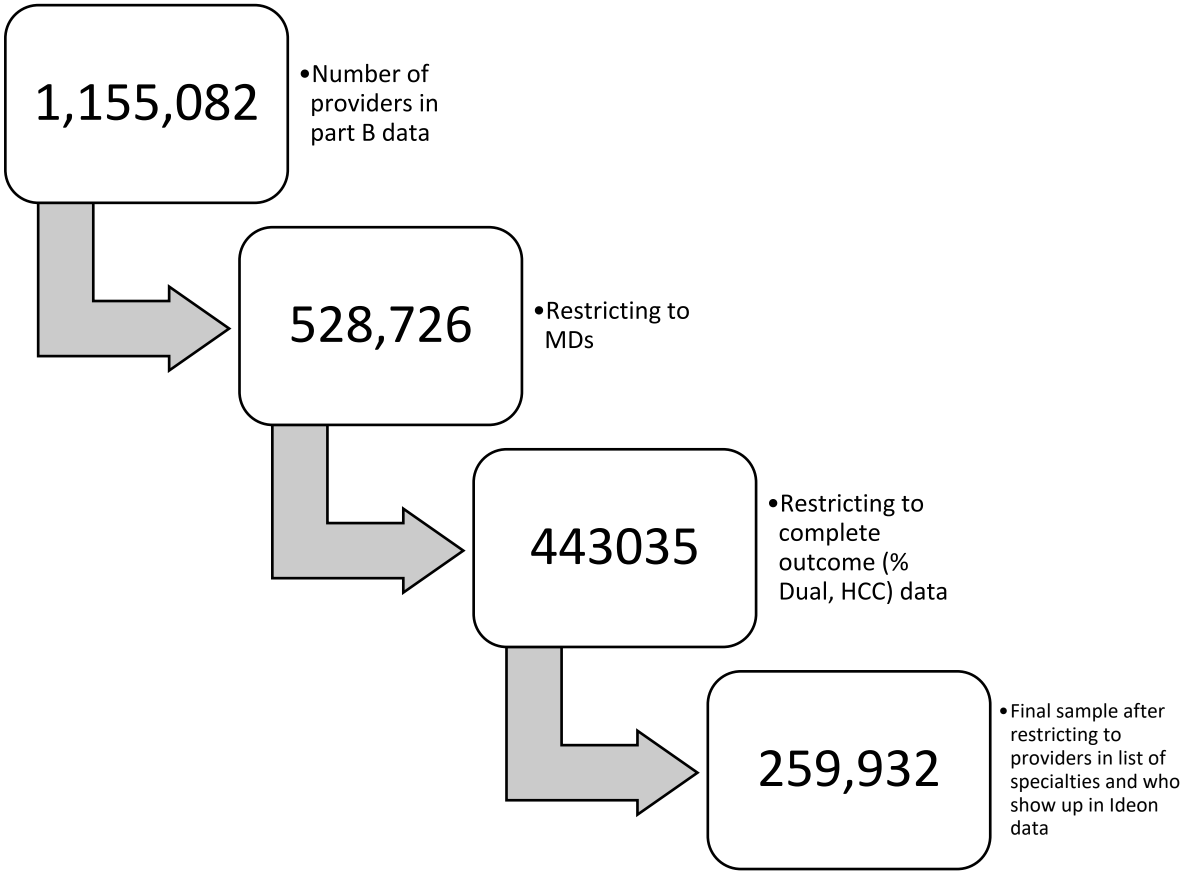

© 2023 Gong JH et al. *JAMA Health Forum.*

eTable 1. Association of Patient Dual and Social Risks with MA Inclusion Rate and In Network Enrollee Proportion by Rurality

Social Risk Model					Clinical Risk Model				
Dual-Eligible Caseload, %	MA Inclusion Rate, %				Mean Beneficiary HCC Score	MA Inclusion Rate, %			
Quintile (Range)	Urban	*P* Value	Rural	*P* Value	Quintile (Range)	Urban	*P* Value	Rural	*P* Value
Q1 (0.0-10.0)	Reference		Reference		Q1	Reference		Reference	
Q2 (10.1-17.4)	-0.1 (-0.3 to 0.0)	.07	-0.8 (-1.6 to 0.0)	0.05	Q2	0.1 (0.0 to 0.3)	.08	0.8 (0.2 to 1.5)	.01
Q3 (17.5-26.2)	-1.8 (-1.9 to -1.6)	<.001	-2.4 (-3.3 to -1.6)	<.001	Q3	-0.4 (-0.6 to -0.2)	<.001	-0.7 (-1.6 to 0.1)	.07
Q4 (26.3-39.2)	-3.8 (-4.0 to -3.7)	<.001	-5.7 (-6.6 to -4.8)	<.001	Q4	-3.1 (-3.3 to -2.9)	<.001	-5.5 (-6.6 to -4.5)	<.001
Q5 (>39.2)	-4.3 (-4.5 to -4.1)	<.001	-9.8 (-10.9 to -8.7)	<.001	Q5	-5.1 (-5.3 to -4.9)	<.001	-7.5 (-8.8 to -6.3)	<.001
Social Risk Model					Clinical Risk Model				
Dual-Eligible Caseload, %	In Network Enrollee Proportion, %				Mean Beneficiary HCC Score	In Network Enrollee Proportion, %			
Quintile (Range)	Urban	*P* Value	Rural	*P* Value	Quintile (Range)	Urban	*P* Value	Rural	*P* Value
Q1 (0.0-10.0)	Reference		Reference		Q1 (0.45-1.13)	Reference		Reference	
Q2 (10.1-17.4)	0.1 (-0.2 to 0.4)	.43	-0.5 (-1.7 to 0.8)	.47	Q2 (1.14-1.36)	-0.2 (-0.5 to 0.1)	.26	0.2 (-0.8 to 1.2)	.64
Q3 (17.5-26.2)	-2.6 (-3.0 to -2.3)	<.001	-2.0 (-3.3 to -0.6)	.004	Q3 (1.37-1.75)	-1.9 (-2.3 to -1.5)	<.001	-2.7 (-3.9 to -1.5)	<.001
Q4 (26.3-39.2)	-6.2 (-6.6 to -5.9)	<.001	-5.9 (-7.5 to -4.4)	<.001	Q4 (1.76-2.43)	-8.3 (-8.8 to -7.9)	<.001	-12.0 (-13.5 to -10.4)	<.001
Q5 (>39.2)	-6.0 (-6.5 to -5.6)	<.001	-9.3 (-11.3 to -7.3)	<.001	Q5 (>2.43)	-13.4 (-13.8 to -12.9)	<.001	-17.4 (-19.3 to -15.5)	<.001

© 2023 Gong JH et al. *JAMA Health Forum.*

eTable 2. Sensitivity Analysis removing potential mediators from the analysis.

Outcome: MA Inclusion Rate					
Dual-Eligible Caseload, %			Mean Beneficiary HCC Score		
Quintile (Range)	MA Inclusion Rate, %[b]	*P* value	Quintile (Range)	MA Inclusion Rate, %[c]	*P* value
Q1 (0.0-10.0)	Reference		Q1 (0.45-1.14)	Reference	
Q2 (10.1-17.4)	-0.3 (-0.5 to -0.2)	<0.001	Q2 (1.15-1.37)	-0.2 (-0.4 to -0.0)	0.24
Q3 (17.5-26.2)	-1.5 (-1.6 to -1.3)	<0.001	Q3 (1.38-1.75)	-1.1 (-1.3 to -0.9)	<0.001
Q4 (26.3-39.2)	-3.2 (-3.4 to -3.0)	<0.001	Q4 (1.76-2.43)	-4.7 (-4.9 to -4.5)	<0.001
Q5 (>39.2)	-3.1 (-3.3 to -2.9)	<0.001	Q5 (>2.43)	-7.0 (-7.2 to -6.7)	<0.001
Outcome: In-Network Enrollee Proportion					
Dual-Eligible Caseload, %			Mean Beneficiary HCC Score		
Quintile (Range)	In Network Enrollee Proportion, %[b]	*P* value	Quintile (Range)	In Network Enrollee Proportion, %[c]	*P* value
Q1 (0.0-10.0)	Reference		Q1 (0.45-1.14)	Reference	
Q2 (10.1-17.4)	-1.4 (-1.7 to -1.1)	<0.001	Q2 (1.15-1.37)	-1.3 (-1.7 to -1.1)	<0.001
Q3 (17.5-26.2)	-4.0 (-4.4 to -3.7)	<0.001	Q3 (1.38-1.75)	-4.1 (-4.5 to -3.8)	<0.001
Q4 (26.3-39.2)	-7.9 (-8.3 to -7.5)	<0.001	Q4 (1.76-2.43)	-12.3 (-12.7 to -11.9)	<0.001
Q5 (>39.2)	-6.9 (-7.4 to -6.4)	<0.001	Q5 (>2.43)	-18.7 (-19.1 to -18.2)	<0.001

Notes: The clinical and social risk models in this table are the same as in the primary manuscript except they do not adjust for rurality or number of years in practice.

© 2023 Gong JH et al. *JAMA Health Forum.*

eTable 3. Sensitivity analysis by minimum number of TM beneficiaries

Dual-Eligible Caseload, %						Mean Beneficiary HCC Score					
MA Inclusion %											
Quartile	No Minimum	50	100	150	200	Quartile	No Minimum	50	100	150	200
Q2	0	-0.1	-0.1	0	0	Q2	0.2	0	-0.1	0	0
Q3	-0.9	-1.2	-1.1	-1.1	-1.2	Q3	-0.5	-0.7	-1	-0.8	-0.6
Q4	-2.5	-2.8	-2.8	-2.9	-3.2	Q4	-3.6	-3.8	-4.6	-4.1	-4.2
Q5	-2.9	-3.2	-3	-3	-3.1	Q5	-5.5	-5.7	-7.5	-6.2	-6.3
In Network Enrollee Proportion											
Quartile	No Minimum	50	100	150	200	Quartile	No Minimum	50	100	150	200
Q2	0	-0.6	-0.6	-0.5	-0.5	Q2	-0.2	-0.5	-0.9	-0.8	-0.7
Q3	-2.5	-3.2	-2.9	-2.9	-3.1	Q3	-2.4	-2.8	-3.3	-2.9	-2.8
Q4	-6.2	-6.9	-6.7	-7	-7.4	Q4	-9.4	-9.8	-11.2	-10.5	-10.5
Q5	-6.4	-7	-6.5	-6.4	-6.5	Q5	-14.8	-15.3	-18.7	-16.3	-16.2

Notes: These models use the same specifications as in the manuscript except use varying minimum beneficiary accounts for inclusion in the analysis.

© 2023 Gong JH et al. *JAMA Health Forum.*
