# Supplementary material for: Proportion of Physicians Who Treat Patients With Greater Social and Clinical Risk and Physician Inclusion in Medicare Advantage Networks
Source: JAMA Health Forum. 2023 Jul 21;4(7):e231991. doi: 10.1001/jamahealthforum.2023.1991 (PMC10362476; doi:10.1001/jamahealthforum.2023.1991)
Supplement: Supplement 2. — Data Sharing Statement [file jamahealthforum-e231991-s002.pdf]

Data Sharing Statement

Gong. Proportion of Physicians Who Treat Patients With Greater Social and Clinical Risk and Physician Inclusion in Medicare Advantage Networks. *JAMA Health Forum*. Published July 21, 2023. doi:10.1001/jamahealthforum.2023.1991

Data
Data available: No

Additional Information
Explanation for why data not available: The data is only availible under a CMS DUA.
